# FPGA-Based Degradation and Reliability Monitor for Underground Cables

**DOI:** 10.3390/s19091995

**Published:** 2019-04-28

**Authors:** Unai Garro, Eñaut Muxika, Jose Ignacio Aizpurua, Mikel Mendicute

**Affiliations:** 1 Faculty of Engineering, Mondragon Unibertsitatea, 20500 Arrasate - Mondragon, Spain; emuxika@mondragon.edu (E.M.); mmendikute@mondragon.edu (M.M.); 2 Faculty of Engineering, University of Strathclyde, Glasgow G11XW, UK; jose.aizpurua@strath.ac.uk

**Keywords:** reliability estimation, cable insulation, Field Programmable Gate Arrays (FPGA), monitoring

## Abstract

The online Remaining Useful Life (RUL) estimation of underground cables and their reliability analysis requires obtaining the cable failure time probability distribution. Monte Carlo (MC) simulations of complex thermal heating and electro-thermal degradation models can be employed for this analysis, but uncertainties need to be considered in the simulations, to produce accurate RUL expectation values and confidence margins for the results. The process requires performing large simulation sets, based on past temperature or load measurements and future load predictions. Field Programmable Gate Arrays (FPGAs) permit accelerating simulations for live analysis, but the thermal models involved are complex to be directly implemented in hardware logic. A new standalone FPGA architecture has been proposed for the fast and on-site degradation and reliability analysis of underground cables, based on MC simulation, and the effect of load uncertainties on the predicted cable End Of Life (EOL) has been analyzed from the results.

## 1. Introduction

Underground cables are subject to varying loads and ambient conditions that progressively degrade their insulation, reducing their RUL. To guarantee the correct behavior of the system, preventive maintenance procedures are performed on the cables before their insulation completely degrades. When the cables are part of a dependable electric power distribution system, reliability-centered maintenance strategies are adopted, and accordingly, it is required that maintenance procedures are performed before the reliability level is below a threshold with certain confidence margin [1,2,3]. However, the reliability estimation of cables is intricate, and an inaccurate health estimation can lead to a costly earlier-than-necessary cable replacement, or to their failure before the predicted time.

An accurate measurement of the degradation level of the cable frequently requires the cable to be disconnected and the system to be put off-line, or in reduced operation mode [4]. However, it is desirable that the monitoring is performed in a non-intrusive manner. In order not to have the system down or providing limited service for long periods of time and guarantee its reliability, the degradation level can be estimated indirectly by monitoring more accessible physical parameters. Parameters can include partial discharges, temperature, tangent delta, leakage currents, permittivity, or current measurements [4,5,6].

Unless direct measurements of the current degradation level can be performed on the cable, the present cable degradation state must be estimated from past measurements and the uncertainties must be considered in this process. Then in the prognosis phase, future forecasted data is employed to predict the future evolution of the cable degradation, either based on physical models or existing data [7].

In this context, the main objective of this article is performing the online estimation of the RUL of a cable, given a reliability requirement, past load measurements, forecast load patterns, and a grid model. An architecture is developed for the cable condition monitoring and prognosis of the cable that performs a quasi-real time execution of thousands to millions of simulations, to evaluate the cable failure Probability Density Function (PDF).

### 1.1. Existing Prognosis Methods

Some of the existing condition monitoring methods employ the online detection and analysis of partial discharges that appear when the cable is relatively close to its EOL. Techniques to identify the condition from partial discharges include fuzzy logic [8], contourlets [9], or the evaluation of the cumulative effect of certain features [10]. However, the methods do not permit performing a long-term cable lifetime estimation.

Reflectometry can also be employed for non-intrusive monitoring of the current degradation state of the cable [11]. However, currently the method requires the disconnection of the cables to install instrumentation [4], and the results do not provide the RUL that depends on the future usage load patterns of the cable.

Some authors have taken the approach to monitor features in the partial discharge signals and predict their accumulated degradation effect and the cable failure time [10]. Wavelet packet analysis data has been employed with neural networks to identify similar transients and predict the failure time [12]. However, the methods were designed to detect incipient failures, rather than performing longer term prognosis. Particle Filters (PFs) have been employed for the condition monitoring of transformers [13], and they have also been employed for performing reliability analysis and prognosis in other fields [14]. In this approach, the behavior described by the particles is extrapolated for the prognosis stage from past behavior with no new input. However, a cable in an electrical grid is subjected to time-varying conditions and parameters that cause deviations and variable statistics in the future behavior. Models that represent the time-dependency of the parameters can be employed for the prognostics and reliability analysis of equipment [15]. These models are often analyzed by means of large MC simulations of the grid conditions that consider complex or very rare events [16,17]. The MC approach permits considering the thermal transients caused by these rare events, which has been shown to be critical to estimate the degradation state of the cable insulators [18].

When Cross-Linked Polyethylene (XLPE) cables are properly insulated from humidity electro-thermal stress is considered the main degradation factor [18]. Consequently, monitoring equipment is put in place, to perform current or temperature measurements and estimate the degradation levels of the cables [19]. Multiple polymer degradation models exist that estimate the condition of the insulation of the cables, including Arrhenius, Zhurkov, and Crine models [20,21,22,23].

Degradation models based on laboratory samples have been scaled statistically to full-sized cables [18] and the Inverse Power Model (IPM) has been validated for estimating the degradation of the insulation of a cable [18,19,24,25,26,27,28], and for the prediction of the cable RUL, by estimating future load patterns [29]. Laboratory-based cable lifetime predictions based on average temperature and load values have been compared and validated in real installation cable lifetime measurements [25]. However, it has been shown that neglecting thermal transients can greatly affect the predicted lifetimes [18]. Considering the thermal transients when performing the prognosis for obtaining the cable RUL can be complex, because frequently the loading of a cable is subjected to fluctuations that are caused by the switching of the electric distribution system.

Additionally, the time-to-failure results obtained by the existing cable degradation models are deterministic, i.e., they estimate a single failure time value without uncertainty information. However, cable operation and degradation parameters are surrounded by different sources of uncertainty, such as the expected load, system usage, variability of the installation parameters, and possible measurement or modeling errors that affect the reliability analysis and the associated maintenance strategy. Load measurements are affected by the accuracy of sensors, and future load predictions are subject to prediction errors and deviations caused by the time-varying grid conditions. Therefore, for dependable cable systems, it is necessary to consider uncertainties and to estimate confidence margins for the RUL results.

The reliability analysis requires performing a probabilistic analysis of the cable failure time, based on the physical models. However, the degradation models are non-linear, and the statistics can be complex to solve analytically. Some authors have approached the case of aerial cable degradation models by means non-sequential MC simulations [30]. However, the statistical sampling of the non-linear degradation processes of underground cables subject to grid events (e.g., faults, switching or maintenance procedures) is complex due to the non-standard probability distributions and time-dependent failure mechanisms. Sequential MC methods are suited to generate those samples if the physical model itself is included within the simulated model [28]. In this context, some variants of the Petri Net formalism such as Stochastic Activity Network (SAN) or Generally Distributed Transition Stochastic Petri Net (GDT_SPN) networks permit including transitions of any probability distribution type [31,32]. The models based on these formalisms can be simulated to estimate the cable reliability values, if the distribution samples for the cable can be generated, e.g., by means of a hybrid statistical-physical model.

Embedded systems based on Digital Signal Processor (DSP) have been employed for online monitoring purposes [33]. However, the simulation of such Petri variant networks with complex physical models in an embedded system Central Processing Unit (CPU) for condition monitoring can be slow and complex considering the computational resources required for the simulation. Additionally, the simulation of the thermal models of underground cables is challenging due to the non-linear response and the long time required to reach the steady-state [34]. This time can cover hundreds of thousands of samples. Therefore, the simulation involves calculating the convolution of those samples. This operation would require DSP and Graphics Processing Unit (GPU) to process data at trillions of floating-point operations per second, including the CPU to GPU data transfer times. FPGAs can be customized for the fast computation of specific algorithms achieving similar or higher performance at lower consumption [35]. While DSP can be employed to perform array operations in an efficient manner, the thermal superposition of hourly thermal transients covering several months to years of thermal simulation can be costly in terms of simulation time and resources. Even high-end computer systems can take days or months to simulate the thousands to millions of simulations required to accurately represent long-term statistics of low failure probability equipment [16]. GPU implementations require careful planning for subprocess division of each different grid model to be simulated efficiently, and they require large memory resources for memory transfer between the separate subprocesses. FPGAs in the other hand permit allocating the necessary resources for implementing the grid model and cable thermal process models, and for performing all thermal superpositions in parallel. The parallelization can be performed down to each single arithmetic block in the process, in a pipelined architecture that permits achieving higher throughputs, reducing the simulation time to a fraction of the times required by common DSP or CPU. Some authors have already proven the use of FPGA for the acceleration of the monitoring of underground cables and other complex processes [36].

In this context, this article proposes the use of an FPGA-based embedded architecture for the accelerated reliability monitoring and prognosis of an underground cable. A novel architecture is presented for the FPGA simulation of the cable electro-thermal degradation model entirely in hardware logic. Methods for adapting the non-linear thermal and degradation models into a pipelined FPGA architecture have been developed. The new architecture can perform the on-site evaluation of the present and future reliability of the cable considering past load measurements and future load pattern predictions by integrating within a hardware SAN MC simulator that simulates the power distribution grid. The architecture performs the estimation of the cable failure time, including confidence margins, with reduced processing times in comparison to software-based simulations.

### 1.2. Organization of the Paper

The rest of this paper is organized as follows: Section 2 describes the thermal heating and degradation models employed; Section 3 describes the adapted models and the architecture that performs the accelerated simulation of the physics of the thermal behavior and degradation of the cables, within a hardware-based SAN simulator; Section 4 describes its application and adaptation process to a specific cable configuration analysis; Section 5 analyzes the performance of the developed architecture and evaluates the results obtained for the given cable set; Section 6 discusses the results obtained; and finally Section 7 summarizes the conclusions obtained.

## 2. Thermal and Degradation Models of Medium and High Voltage Cables

The degradation process of a cable insulation within an electrical grid follows a time-varying state-space model that depends on the present and past applied electric field and temperatures. The Zhurkov-based electro-thermal degradation model has been adopted for this article [37]. Mazzanti showed that the lifetime equation from laboratory measurements based on this model can be scaled statistically to a full-sized cable as [18]

(1) $$\tau (\vartheta ,E)={\left(-\frac{ln(1-P_{D})}{D}\right)}^{\frac{1}{\beta_{t}}}\alpha_{\tau ,0}e^{\frac{w-\chi E}{R\vartheta }}$$

where τ(ϑ,E) is the lifetime of the cable when an electric field *E* and an absolute temperature ϑ are applied to the cable insulator, *w* is the activation energy of the insulator destruction process, χ is a structural parameter, $P_{D}$ is the design failure probability, $\alpha_{\tau ,0}$ is the scale parameter, *D* is the enlargement factor, $\beta_{t}$ is the shape parameter of the Weibull cable time-to-failure probability distribution, and *R* is the universal gas constant.

The fraction of cable lifetime loss $\Delta \gamma_{i}$ at time interval *i* for a time duration of *h* (assuming that *h* is small enough to consider that both thermal and electrical stresses are constant) can be approximated by an IPM model as [18]

(2) $$\Delta \gamma_{i}\approx \frac{h}{\tau (\theta_{i},E_{i})}$$

where $E_{i}$ and $\theta_{i}$ are the electric field and temperature applied to the cable insulator at time interval *i*.

In this context, the total lifetime loss can be estimated from the continuous estimation of the insulator temperature and electric field.

The thermal heating of the cable depends on the power dissipated both within the cable under analysis and within the adjacent cables. The thermal model adopted in IEC60853-2 [38] has been validated by several authors [26,29,39] for modeling the temperature evolution of the cable insulator. In this model, the thermal temperature rise θ(t) above ambient temperature is calculated as [38]

(3) $$\begin{array}{c} \theta \left(t\right)=\theta_{s}\left(t\right)+\alpha \left(t\right)\theta_{e}\left(t\right)+\alpha \left(t\right)\sum_{k}\theta_{pk}\left(t\right) \end{array}$$

where $\theta_{s}\left(t\right)$ is self-heating temperature rise caused by the total power dissipated within the cable of interest, $\theta_{e}\left(t\right)$ is the temperature rise caused by influence of the soil, $\theta_{pk}\left(t\right)$ is the temperature rise caused on the cable of interest *p* by the total power dissipated in each adjacent cable *k*, and α(t) is the attainment factor for the transient temperature rise between the conductor and the outer surface [34].

The self-heating process is modeled using an equivalent RC ladder circuit that can be approximated with a reduced (two loop) circuit. The thermal response can then be represented by a second order Laplace transfer function H(s) [34]

(4) $$\begin{array}{cc} & L\left\{\theta_{s}\left(t\right)\right\}=\Theta_{s}\left(s\right)=H\left(s\right)W_{c}\left(s\right) \end{array}$$

(5) $$\begin{array}{cc} & H\left(s\right)\approx \frac{T_{A}+T_{B}+\left(Q_{B}T_{A}T_{B}\right)s}{Q_{A}Q_{B}T_{A}T_{B}s^{2}+\left(Q_{A}\left(T_{A}+T_{B}\right)+Q_{B}T_{B}\right)s+1} \end{array}$$

where $W_{c}\left(s\right)$ is the Laplace transform of the power dissipated in the main cable conductor, and $T_{A},$ $T_{B},$ $Q_{A}$ and $Q_{B}$ are the thermal resistivity and capacitances of the equivalent thermal circuit.

The transient thermal response to a load step due to the effect of the soil can be calculated as [38]

(6) $$\theta_{e}\left(t\right)=W_{t}\frac{\rho_{s}}{4\pi }\left[-\mathrm{Ei}\left(-\frac{D_{e}^{2}}{16\delta_{s}t}\right)+\mathrm{Ei}\left(-\frac{L_{d}^{2}}{\delta_{s}t}\right)\right]$$

where $W_{t}$ is the total power dissipated in the cable including sheath and armor losses, $\rho_{s}$ and $\delta_{s}$ are the thermal resistivity and diffusivity of the soil, $D_{e}$ is the external cable diameter, $L_{d}$ is the depth at which the cable is laid, and Ei is the exponential integral function, defined as

(7) $$\mathrm{Ei}\left(x\right)=-\int_{-x}^{\infty }\frac{e^{-t}}{t}\;dt$$

Finally, the thermal response to a mutual heating caused by each adjacent cable *k* on the cable *p* can be calculated by

(8) $$\theta_{pk}\left(t\right)=W_{t}\frac{\rho_{s}}{4\pi }\left[-\mathrm{Ei}\left(-\frac{d_{pk}^{2}}{4\delta_{s}t}\right)+\mathrm{Ei}\left(-\frac{d_{pk}^{\prime 2}}{4\delta_{s}t}\right)\right]$$

where $d_{pk}$ and $d_{pk}^{\prime }$ are the distance to the adjacent cable and its image, respectively.

For varying loads, the principle of superposition is employed [34], and the variation of electrical resistivity of the conductors with temperature needs to be considered by correcting the results with the upper bound defined in [40] as adopted by the IEC60853 standard [38].

## 3. A Novel FPGA Structure for Cable Reliability Estimation

**Figure 1:**
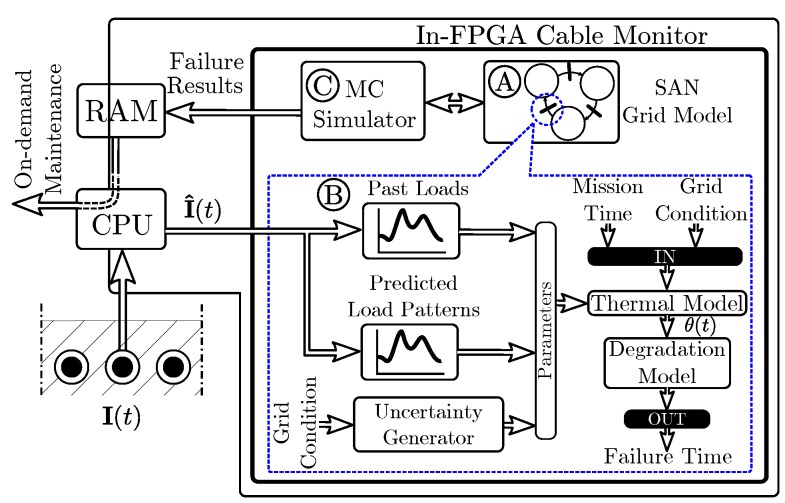
FPGA-based cable reliability monitor architecture.

Figure 1 shows the proposed FPGA-based cable reliability monitor. The monitor estimates the degradation process of a cable within a grid, subject to varying load and thermal conditions. The architecture consists of A a SAN model for the grid switching mechanisms and grid failure logic; B transition or activity blocks within the model that simulate the cable degradation for generating its failure statistics; and C a MC simulator that simulates the model in hardware and records the results directly in Random Access Memory (RAM). In this architecture, the CPU is only employed to load the simulation conditions and input data, and to post-process the simulation results. The input data includes past current measurements, predicted load values $\hat{I}\left(t\right)$, uncertainty deviation parameters, and the number of simulations to be performed. The FPGA runs the simulations without the aid of the CPU and records the time of failure in each simulation directly in RAM through Direct Memory Access (DMA). For diagnostic purposes, past temperature measurements can be used as input if available. For the evaluation of the future degradation trajectory, predicted future load patterns and a grid switching model are required.

### 3.1. Pipelining of the Thermal Model

The hardware implementation permits accelerating the simulation of the sequential and repeating thermal process by through the parallelization of each subprocess. Two approaches can be taken when dividing each process: (a) blocks with reduced latency; (b) blocks that are pipelined for high throughput with generally larger latency.

In the non-pipelined approach, the subprocesses of a thermal response with a mission time of *N* samples require $N\;\cdot \;\tau_{d}$ clock cycles to perform the simulation, where $\tau_{d}$ is the block delay or *latency* of the block in clock cycles.

**Figure 2:**
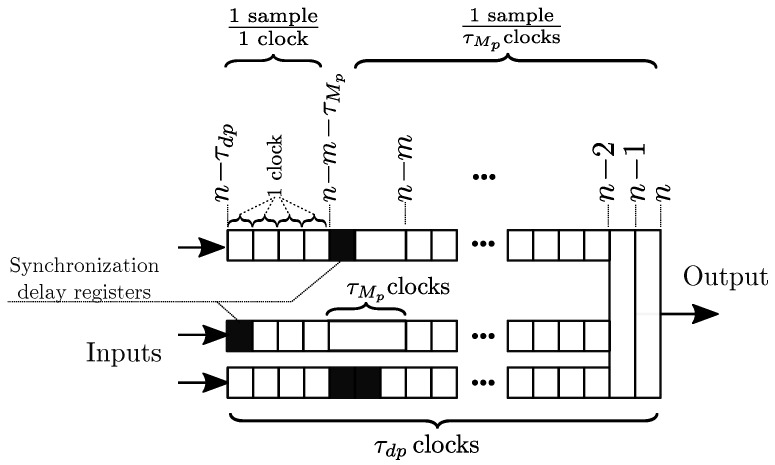
Pipeline block latencies and synchronization.

The pipelined approach divides the process into multiple sub-blocks of reduced complexity and has generally a larger $\tau_{dp}$ total latency as shown in Figure 2. However, the throughput is defined by the sub-block with the largest latency $\tau_{Mp}$, which is ideally a single clock cycle. Sub-blocks after this block will only be able to produce results at 1 sample per $\tau_{Mp}$ clocks. In total, the simulation requires $\left(N-1\right)\tau_{Mp}+\tau_{dp}$ clock cycles. Therefore, long-term simulations where the mission time *N* is large, benefit from pipeline implementations where $\tau_{Mp}$ is reduced, and the method has been adopted for this design.

To produce the maximum possible throughput, in this implementation each arithmetic operator has either a total latency of 1 clock, or they are fully pipelined arithmetic blocks to achieve a maximum pipeline block latency of $\tau_{Mp}=1\;\mathrm{clock}$. Therefore, producing a 1 sample/clock throughput.

Floating-point implementations in hardware logic are large and cause increased latencies, whereas fixed-point blocks are reduced in size and simple to extend for larger precision values. Therefore, each arithmetic block employs fixed-point arithmetic.

The required resolution in each stage in the pipeline varies with the accuracy requirement for the output in each stage. The range and resolution required for the input and for each of the operators was established according to range and precision of the output. Due to this varying resolution required in each arithmetic operation in the pipeline, for all the diagrams in the paper, arithmetic blocks need to adapt their input values and output values to the data sizes and resolutions of the corresponding variables. Therefore, the blocks in the diagrams include implicit bit-alignment blocks in their inputs and outputs that perform bit shifting and slicing, to align the variables.

The required resolution for each arithmetic block in the pipeline varies depending on the dimensions and characteristics of each cable and grid configuration. Therefore, the implementation should be ideally optimized to fit the dimensions of the variables for the different cable and grid configurations. However, to generalize the implementation, the following variable resolutions were defined in this implementation:

- Currents: a 32-bit data size and 16 fraction bits were employed to represent both short circuit currents and the required uncertainties.
- Temperatures: a 24-bit data size and 16 fraction bits were employed to represent self-heating, mutual heating, and soil effect processes. However, the intermediate variables have varying sizes, due to the varying coefficient values in each cable model. For the shown use case, up to 73-bit multiplier registers were required.
- Degradation: a 32-bit data size with a 31-bit fraction value is employed, due to the exponential curves in the Zhurkov-based degradation model Equation (1).

### 3.2. SAN Model of the Grid

The thermal model is governed by the instantaneous condition of the grid, which is modeled as a SAN network. SAN networks are a variant of Petri Nets [41] that in addition of stochastic intervals with any probabilistic distribution, also add the concept of *input and output gates*, which can operate complex functions to describe the behavior of any system.

**Figure 3:**
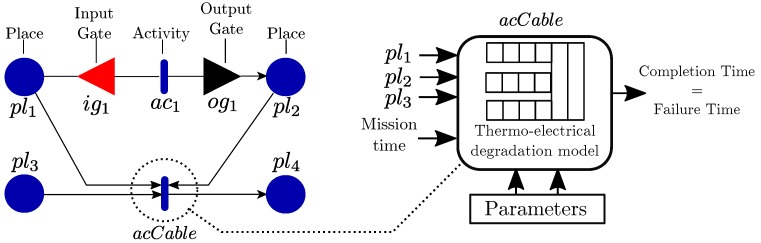
SAN model example with a cable thermo-electrical degradation model activity integrated within the model.

SAN models consist of *places*, *activities*, *input gates*, and *output gates* as shown in Figure 3. The state of the model is represented by a $\mu_{i}$ marking for each ith place $pl_{i}$. Input and output gates include *functions* that alter the markings of the connected places when the activity is triggered. When gates are not drawn as in *acCable* activity in Figure 3, default gates are implicit, where their behavior is the same as in Petri Nets where marks are removed from the input places and added to the output paces. Activities govern the timing of events in a SAN model by defining stochastic, deterministic, or instant times at which they *complete* by triggering input and output gates, generating a new model marking μ. Input gates define conditions that enable activities by defining an *enabling predicate* function. When all enabling predicates are met, an activity becomes *active* and a new completion time is generated.

In this architecture, the SAN model simulator is integrated in the FPGA, and it controls the inputs of the thermal simulation. The thermal and degradation simulator pipeline itself is integrated as an activity inside the SAN model (*acCable* in Figure 3). This activity implements the pipelined logic that simulates the thermal behavior of the cable and evaluates the instantaneous degradation of the insulator, until its failure time. The pipelined model simulates the thermal process under the conditions defined by the connected places ($pl_{1}$, $pl_{2}$ and $pl_{3}$ in Figure 3). The connected places can indicate different load conditions, switching, or maintenance downtimes that affect the cable model behavior.

**Figure 4:**
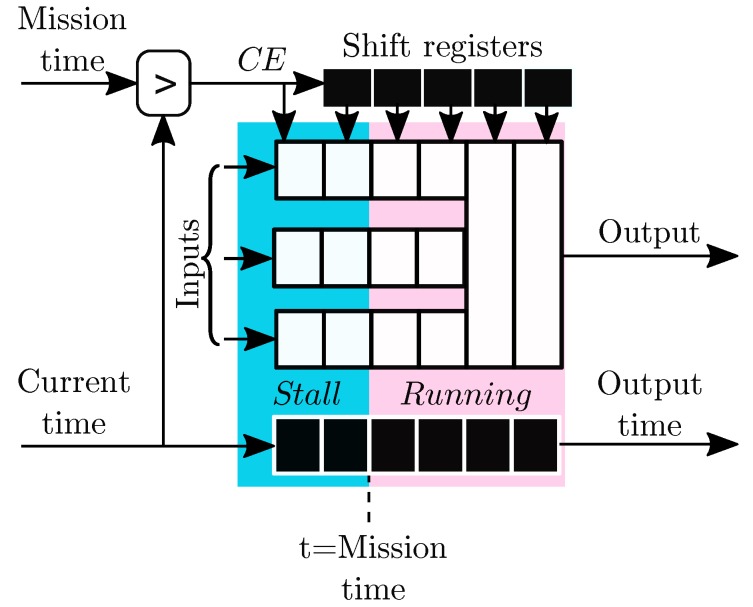
Pipeline stall during SAN model state changes.

Therefore, the cable model pipeline needs to attend to grid state changes in the SAN model. The SAN model simulator evaluates the time at which a state change will be occurring in the grid and defines a *mission time* for the thermal simulation to end. The simulation is run until this mission time. When this time is reached, no new input is available (a data *hazard* condition occurs in the pipeline) and parts of the pipeline need to be disabled (*pipeline stall*). The rest of the pipeline that has valid queued data continues processing its output until the last value in the pipeline is processed and the new cable condition is calculated as shown in Figure 4. The pipeline stall is controlled by a Clock Enable (CE) signal that is carried through the whole pipeline. Therefore, in all the blocks designed for this implementation, a global CE signal and a time value are carried through the pipeline, indicating the validity and the sample time of each value. When the mission time or the cable failure condition is reached, the thermal model activity issues its completion time as output. A time signal is also carried through shift registers in the pipeline as shown in Figure 4. These time signals are not included in the thermal model diagrams for readability purposes.

The following subsections describe the hardware logic implementation of the models for each thermal and degradation process.

### 3.3. Self-Heating Process

For currents modeled as a staircase signal, an accurate thermal response of Equation (4) can be evaluated by discretizing the transfer function Equation (5) employing the zero-order hold method, with the same sample rate as that of the current measurements. The discretization results in a second order discrete Z transform function

(9) $$H\left(z\right)=\frac{Z\{\theta_{s}\left[n\right]\}}{Z\{W_{c}\left[n\right]\}}=\frac{\Theta_{s}\left(z\right)}{W_{c}\left(z\right)}=\frac{C_{m,1}z+C_{m,2}}{z^{2}-C_{d,1}z-C_{d,2}}$$

where $C_{m,1}$, $C_{m,2}$, $C_{d,1}$, and $C_{d,2}$ are constant for each cable model, and $W_{c}\left[n\right]$ and $\theta_{s}\left[n\right]$ are the power dissipated in the conductor and self-heating temperature rise at discrete-time instant *n*, respectively.

The Infinite Impulse Response (IIR) architecture described by Equation (9) requires 4 parallel multiplications and two sequential additions to be performed for each output sample. This structure can prevent the sub-block from operating at high clock frequencies, which is desirable for a high throughput pipeline. To this end, a look-ahead transformation can be employed to increase the latency of the filter by two extra clocks that permit a fully pipelined architecture [42]

(10) $$\frac{\Theta_{s}\left(z\right)}{W_{c}\left(z\right)}=\frac{C_{W,1}z^{3}+C_{W,2}z^{2}+C_{W,3}z+C_{W,4}}{z^{4}-C_{\theta ,3}z-C_{\theta ,4}}$$

where

(11) $$\begin{array}{ccc} C_{\theta ,3} & = & C_{d,1}^{3}+2C_{d,1}C_{d,2} \end{array}$$

(12) $$\begin{array}{ccc} C_{\theta ,4} & = & C_{d,1}^{2}C_{d,2}+C_{d,2}^{2} \end{array}$$

(13) $$\begin{array}{ccc} C_{W,1} & = & C_{m,1} \end{array}$$

(14) $$\begin{array}{ccc} C_{W,2} & = & C_{d,1}C_{m,1}+C_{m,2} \end{array}$$

(15) $$\begin{array}{ccc} C_{W,3} & = & C_{d,1}^{2}C_{m,1}+C_{d,2}C_{m,1}+C_{d,1}C_{m,2} \end{array}$$

(16) $$\begin{array}{ccc} C_{W,4} & = & C_{d,1}^{2}C_{m,2}+C_{d,2}C_{m,2} \end{array}$$

The transfer function in Equation (10) corresponds to the difference equation

(17) $$\begin{array}{c} \Theta_{s}\left[n\right]=\overset{\mathrm{Non}-\mathrm{recursive}\mathrm{part}}{\overset{︷}{C_{W,1}W_{c}\left[n-1\right]+C_{W,2}W_{c}\left[n-2\right]+C_{W,3}W_{c}\left[n-3\right]+C_{W,4}W_{c}\left[n-4\right]}}\\ +\underset{\mathrm{Recursive}\mathrm{part}}{\underset{︸}{C_{\theta ,3}\Theta_{s}\left[n-3\right]+C_{\theta ,4}\Theta_{s}\left[n-4\right]}} \end{array}$$

**Figure 5:**
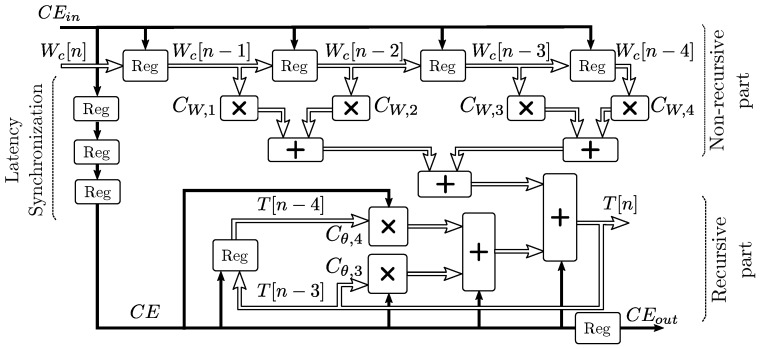
Fully pipelined self-heating model implementation.

Figure 5 shows the FPGA implementation of the IIR filter described by Equation (17). The numerator part results in a non-recursive filter that can be implemented as a direct Finite Impulse Response (FIR) design [42], and the denominator part describes a recursive filter that permits a latency of 3 clocks ($\Theta_{s}\left[n-3\right]$ to $\Theta_{s}\left[n\right]$) to calculate the output. This extra latency permits calculating both multiplications and the two additions at higher clock frequency and therefore higher throughput. Due to the latency inherent to the direct FIR filter implementations, registers have been added delaying the enablement of the recursive filter part, to synchronize the latencies of both parts as shown in Figure 5. Each of the arithmetic blocks executes fixed-point operations in a single clock cycle. The pipeline stall, flush and resume mechanism has been implemented by a shifting clock enable signal CE that pauses the filter recursion when inputs are invalid, while the sub-blocks with valid inputs and time-independent blocks can still operate. The output $CE_{out}$ signal permits synchronization with the rest of the pipelined simulator blocks that operate at different latencies.

### 3.4. Mutual and Soil Heating Processes

The mutual and soil heating processes are calculated from Equations (6) and (8), and the superposition of effects is applied. In software implementations, the exponential integral function is generally approached by a sequential series approximation. However, for hardware logic implementations, the complexity of the divider operators, their sequential operation, and the superposition of the multiple transients covering thousands of hours is impractical due to the incurred latencies and the large resources required. The implementation of the mutual heating and soil effect has been performed in the same manner. Therefore, the following FPGA implementation method described for the mutual heating effect also applies to the soil effect model implementation.

The discrete-time thermal response of the mutual heating process can be defined by the discrete convolution

(18) $$\theta_{pk}\left[n\right]=\Delta W\left[n\right]\times g_{pk}\left[n\right]=\sum_{i=0}^{\infty }\Delta W\left[n-i\right]g_{pk}\left[i\right]$$

where $\theta_{pk}\left[n\right]$ is the discrete temperature rise at discrete-time instant *n* due to the mutual heating, ΔW[n] is the dissipated power increment at discrete-time instant *n*, and $g_{pk}\left[n\right]$ is the unit step response of the mutual heating process. However, due to the long time required for $g_{pk}\left[n\right]$ to reach steady-state conditions, the convolution would require a large size FIR filter involving thousands of multiply-add logic blocks, which is unfeasible in current FPGA technology.

**Figure 6:**
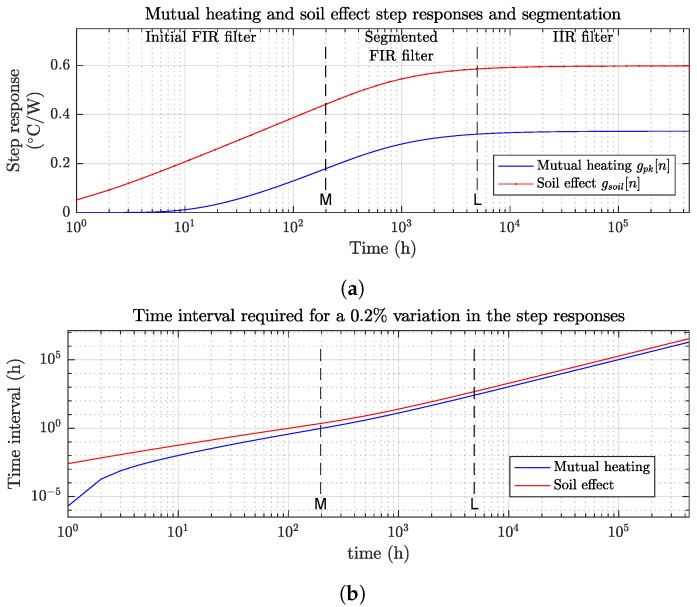
Mutual and soil thermal step responses: (**a**) partitioning of the mutual and soil step responses into separate segments; and (**b**) time interval required for the step response to increase by 0.2%.

The presented architecture takes advantage of the characteristics of the mutual and soil heating processes. The step responses of both thermal processes, described by $g_{pk}\left[n\right]$ and $g_{soil}\left[n\right]$, are shown in Figure 6a. Figure 6b describes the time increment required for a 0.2% change of the step responses. Therefore, if the response curve is divided into approximate sub-segments with constant value, these time intervals would suffice to achieve a 0.2% accuracy. Both the soil and mutual heating processes grow rapidly in the initial transition hours up to *M* hours. After those *M* hours, the growing rate decreases geometrically, and after *L* hours it reaches to a near steady-state condition. However, assuming that $g_{pk}$ is at the steady-state from time instant *L* can cause unacceptable estimation errors for the desired accuracy, even with *L* in the order of thousands of hours.

The proposed architecture divides the $g_{pk}\left[n\right]$ response into 3 segments, as shown in Figure 6a: An initial FIR filter covering the first M = 200–300 h of transition, a segmented FIR filter set covering *L* samples depending on the cable structure, and an IIR filter for the response after that time instant.

**Figure 7:**
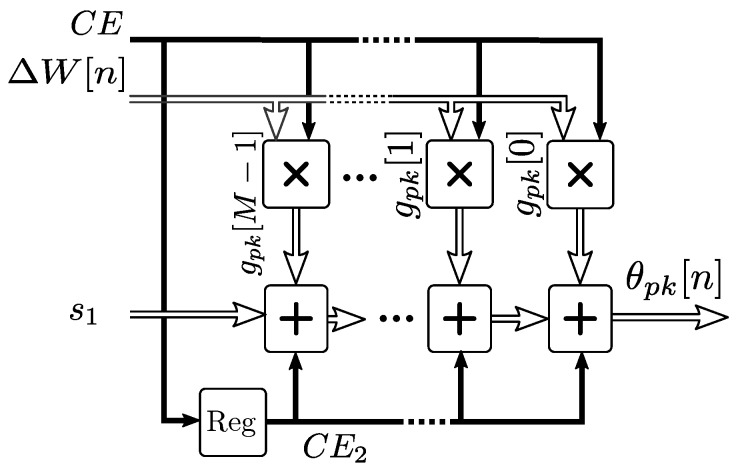
First segment implementation as a transposed FIR filter.

**Figure 8:**
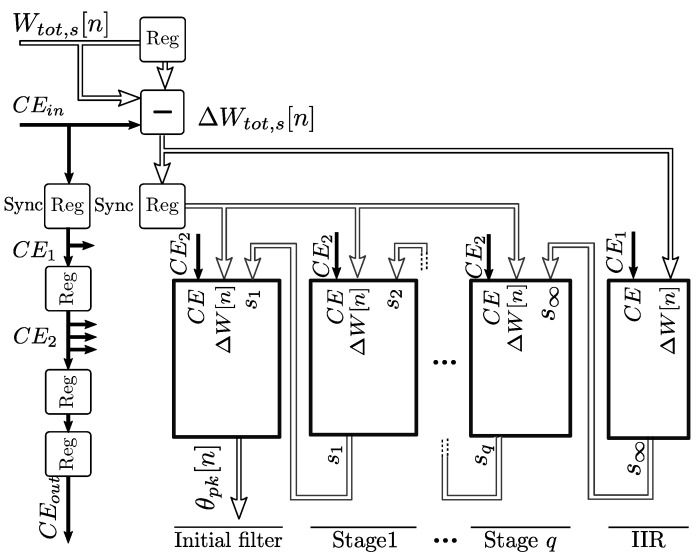
Complete pipelined hardware logic implementation of the mutual heating and soil effect models.

For the first segment FPGA implementation, the synthesis of a direct filter architecture would not be feasible within the given latency constraints, due to its large filter size that makes routing complex. A transposed filter architecture shown in Figure 7 permits pipelining and a two-clock pipeline latency. The initial filter input $s_{1}$ is connected to the *Stage 1* filter output $s_{1}$ of the segmented FIR filter set, as shown in Figure 8.

For the last $g_{pk}$ segment, it was found that after *L* hours of transition time, $g_{pk}\left[n\right]$ can be approximated by a process ${\hat{g}}_{pk,L}\left[n\right]$ described by a first order response plus a step function that reaches to the steady-state condition. Therefore, for n≥L, Equation (8) can be discretized as

(19) $$\begin{array}{cc} g_{pk}\left[n\right] & \approx {\hat{g}}_{pk,L}\left[n-L\right],\;n\geq L \end{array}$$

(20) $$\begin{array}{c} \begin{array}{cc} Z\{{\hat{g}}_{pk,L}\left[n\right]\} & =\frac{g_{pk}\left[L\right]z}{z-1}+\left(g_{pk}\left[\infty \right]-g_{pk}\left[L\right]\right)\frac{z}{z-1}\;\cdot \;\frac{1-e^{-\tau_{s}/\tau_{pk}}}{z-e^{-\tau_{s}/\tau_{pk}}} \end{array} \end{array}$$

where $\tau_{s}$ is the sampling interval and $\tau_{pk}$ is the time constant of the first order response.

**Figure 9:**
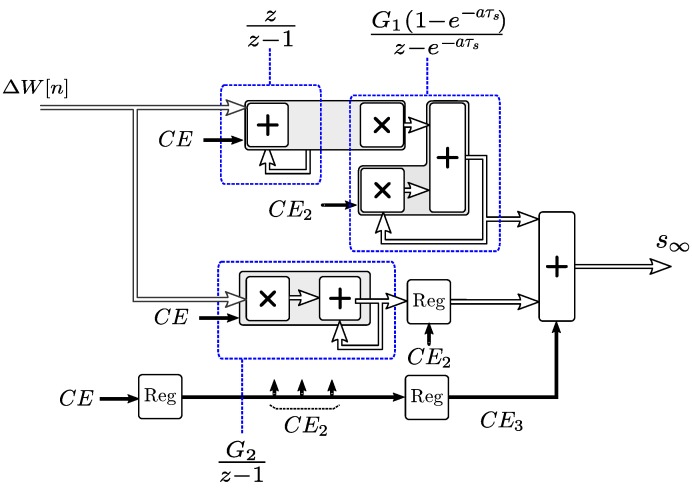
IIR filter implementation for the last segment.

Merging the parts of Equation (20) into a 3rd order transfer function is not suggested, as it can lead to rounding errors and increased latency. Therefore, it has been implemented by obtaining the difference equations of the transfer function in three parts and converting each part into the blocks marked in Figure 9, where $G_{1}=g_{pk}\left[\infty \right]-g_{pk}\left[L\right]$ and $G_{2}=g_{pk}\left[L\right]$, with a total of 3 clock latency.

Accordingly, the convolution in Equation (18) is approximated as

(21) $$\theta_{pk}\left[n\right]\approx \;\;\sum_{i=0}^{L-1}\;\;\;\Delta W\left[n\;-\;\;i\right]g_{pk}\left[i\right]+\;\sum_{i=L}^{\infty }\;\;\;\Delta W\left[n\;-\;\;i\right]{\hat{g}}_{pk,L}\left[i\;-\;L\right]$$

However, the size *L* required for a given accuracy of Equation (20) can be too large to fit in an FPGA. The necessary number of samples *L* is dependent on the time constants defined by the cable dimensions, soil thermal characteristics, and cable setup. Tests showed that for cable setups with large time constants and L=5000 samples, a maximum deviation of 0.4% was achieved by the first order approximation Equation (20) for the mutual heating, and a maximum error of 0.2% for the soil effect.

The implementation of this segmented filter set can be simplified by taking advantage of the decreasing derivative of $\theta_{pk}\left(t\right)$. As shown in Figure 6b, after *M* time samples the number of hours required for the $g_{pk}\left[n\right]$ to increase more than certain resolution limit grows geometrically. Therefore, the filter implementation can be reduced by segmenting $g_{pk}\left[n\right]$ into constant valued filter stages of growing size, where each stage size is calculated for the given accuracy. Each stage is then converted as follows, into simplified sub-filter architectures as shown in Figure 8.

In this implementation, the segmented filter set contains a set of *q* FIR filters that have large $\left\{m_{1}\cdots m_{j}\cdots m_{q}\right\}$ filter lengths, with a constant coefficient value ${\hat{g}}_{pk}^{\left(j\right)}$ for each. This is equivalent to increasing geometrically the sampling time of $g_{pk}\left[n\right]$, while the sampling rate of the input power transitions is maintained. In between $g_{pk}$ samples, filter values are kept constant, and the transition to the steady-state after *L* samples is implemented by the IIR filter $Z({\hat{g}}_{pk,L}\left[n\right])$ described in Equation (20). Therefore,

(22) $$\begin{array}{c} \begin{array}{c} \theta_{pk}\left[n\right]\approx \underset{\mathrm{FIR}}{\underset{⎵}{\sum_{i=0}^{M-1}\;\;\Delta W\left[n\;\;-\;i\right]g_{pk}\left[i\right]}}+\underset{\mathrm{set}\mathrm{of}q\mathrm{FIR}\mathrm{subfilters}}{\underset{⎵}{{\hat{g}}_{pk}^{\left(1\right)}\sum_{i=M}^{M+m_{1}-1}\Delta W\left[n\;\;-\;i\right]\;+\;\ldots \;+\;{\hat{g}}_{pk}^{\left(q\right)}\;\;\;\;\sum_{\begin{array}{c} i=M+m_{1}\\ +m_{2}+\cdots +m_{\left(q-1\right)} \end{array}}^{\begin{array}{c} M+m_{1}+m_{2}\\ +\cdots +m_{q}-1 \end{array}}\;\;\Delta W\left[n\;\;-\;i\right]\;}}+\;\underset{\mathrm{IIR}}{\underset{⎵}{\sum_{i=L}^{\infty }\;\Delta W\left[n\;-\;i\right]{\hat{g}}_{pk,L}\left[i\;-\;\;L\right]}} \end{array} \end{array}$$

**Figure 10:**
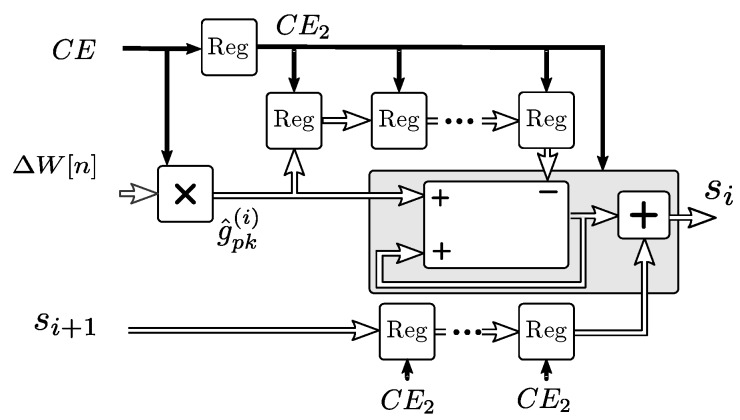
Implementation of each sub-filter in the second segment of the thermal response.

This method permits reducing the *L* multiply-accumulate blocks required for the filter in Equation (21), into an architecture of *q* sub-filters with a single ${\hat{g}}_{pk}^{\left(j\right)}$ multiplier and an accumulator for each stage *j*, as shown in Figure 10. The shift registers in each stage account for the $m_{j}$ ΔW samples that are processed by each jth filter stage at a given time, and are removed when the samples enter the next stage.

The complete model implementation is shown in Figure 8. The initial filter and each stage filter outputs are connected to the next stage filter inputs, and the IIR filter from Equation (20) is connected as the last stage. The total input to output latency is 3 clocks with a throughput of 1 sample per clock for consecutive outputs. Delay registers labeled *sync* permit the synchronization of the outputs due to the differences in latency between the segments.

For this implementation, M=200 was used for the initial filter, *q* = 218 s stage filters were required for a 0.2% accuracy of the mutual heating, and *q* = 114 for the soil effect. If a better resolution is required, a linearly interpolated coefficient set can also be used with extra multiply-accumulate blocks, with the associated increase of the resource requirements.

The resource use can be further reduced by merging both mutual heating and soil effect filters into a single filter set. In this implementation both filters were synthesized separately, for testing, analysis, and validation purposes.

### 3.5. Thermal Degradation Model

**Figure 11:**
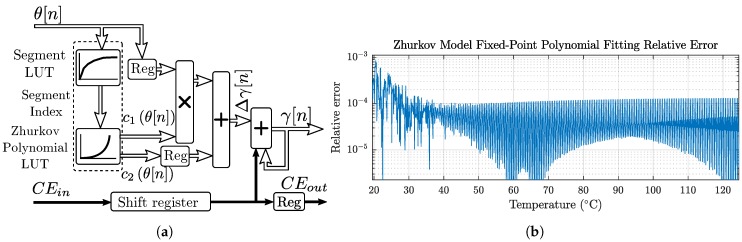
Mutual and soil thermal step responses: (**a**) pipelined Zhurkov model architecture implementation; and (**b**) relative error of the polynomial fitting of the Zhurkov Model.

The degradation function described by Equation (2) grows exponentially with the temperature. A reduced latency implementation was achieved by segmenting the curve and approximating each segment by a linear regression. The implemented architecture is shown in Figure 11a. The temperature θ(n) is input to a first stage Lookup Table (LUT) that indexes the segment. A non-linear segmentation permits increasing the resolution in high derivative areas for better accuracy, and permits reducing the FPGA LUT resources required to implement polynomial indexing and to record their coefficients.

A second LUT obtains the polynomial coefficients $c_{1}$ and $c_{2}$ corresponding to the segment index that approximate Equation (2). The degradation increment Δγ for each time interval of $h=\tau_{s}$ hours, is then estimated as

(23) $$\Delta \gamma \left(\theta ,n\right)\approx \frac{\tau_{s}}{\tau \left(\theta (n)\right)}\approx c_{1}\left(\theta (n)\right)\;\cdot \;\theta \left(n\right)+c_{2}\left(\theta (n)\right)$$

An adder block accumulates the Δγ(θ,n) increments. Fixed-point operators are 44-bit fraction resolution in this implementation, achieving an accuracy of 0.1% as shown in Figure 11b. All blocks except the accumulator are memoryless. Therefore, pipeline clock enablements only affect the adder block.

For simplicity, the electrical field is assumed to be constant for this implementation. A two-dimensional LUT and interpolation can be similarly used for varying electrical field cases.

### 3.6. Currents and Dissipated Power

**Figure 12:**
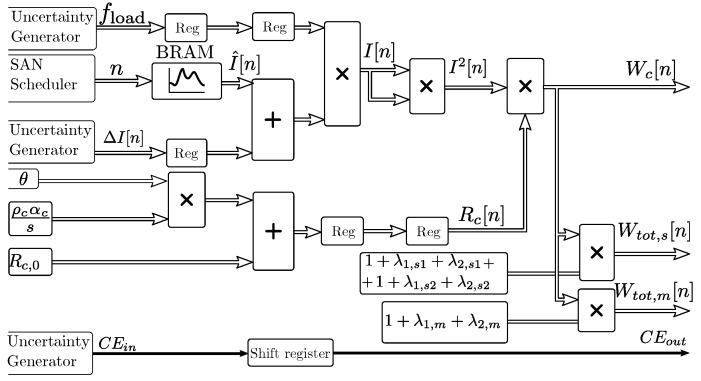
Pipelined input power sequence generation logic.

Figure 12 shows the implementation for the models of currents and dissipated power. The block generates separate output values for the power dissipated in the main conductor $W_{c}$, and the total power dissipated in the middle cable $W_{tot,m}$ and in adjacent cables $W_{tot,s}$. Cable load values are loaded from a Block RAM (BRAM) memory in the programmable logic part of the FPGA, as shown in Figure 12. In this implementation, hourly full-year load values are recorded. The BRAM can be accessed and preloaded by the CPU for each simulation, to include past current measurements and updated predicted future load patterns.

An uncertainty generator adapted for the cable sensor and prediction algorithms as described in Section 4 injects load errors with standard deviations defined in the simulation parameters. The uncertainty generator also monitors the state of the SAN grid model and controls the load factor $f_{load}$ that multiplies the input power. The discrete-time index *n* is driven by the SAN scheduler in the MC simulator, and $CE_{in}$ enables the pipeline while no switching of the grid occurs. Sheath and armor loss factors for the middle cable $\lambda_{1,m}$ and $\lambda_{2,m}$ and for each ith side cable $\lambda_{1,si}$ and $\lambda_{2,si}$, and resistivity $\rho_{c}$, temperature coefficient $\alpha_{c}$, and section values *s* of the conductor are included as multiplying parameters for adapting the model to different cables or varying parameters. The loss factors can be included within the mutual and soil heating model filters described in Section 3.4 for a reduced size implementation, at the cost of a reduced flexibility.

### 3.7. Interfacing and Control by the SAN MC Simulator

The thermal and degradation simulator is integrated as part of the SAN model, as shown in Figure 1. The cable simulation is controlled by two inputs: the partial mission time and the current grid condition. The former indicates the final time to be simulated before any possible grid condition change. The latter includes the switching state for this use case, which can vary the load factor injected to the cable model. Once the partial mission time has been reached and all pipelines have finished processing the queued samples, the accumulator in the degradation block in Figure 11a holds the degradation value of the cable. If the degradation has reached its maximum limit, a *failed* condition and the cable failure time are notified as output.

The simulator then records the failure time results in RAM by DMA. The CPU collects the results and evaluates the failure Cumulative Distribution Function (CDF) and confidence margins. Simulations end after obtaining the required confidence margins.

### 3.8. Diagnostics and Prognosis of the Cable RUL

**Figure 13:**
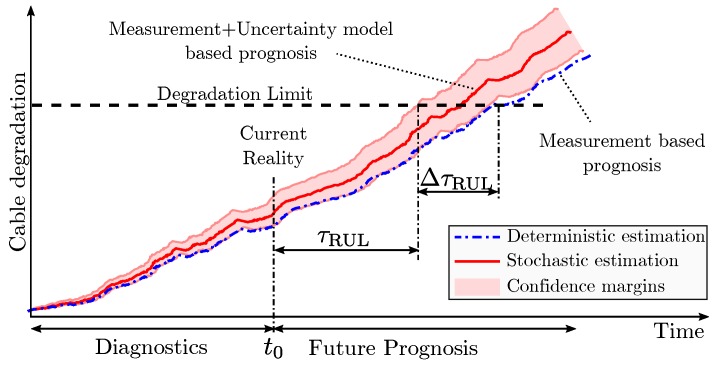
Effect of uncertainties on cable diagnostics and prognosis.

At any given time $t_{0}$ the insulator degradation state can be estimated from past measurements. This diagnostics step is affected by the model errors and measurement deviations. The future predictions are affected by the past monitoring deviations, model errors, and future load estimation algorithm errors. A deterministic analysis with a single simulation cannot represent the future stochastic behavior of the grid, and neither the uncertain errors introduced by the past monitoring and model deviations. The differences in the degradation paths between the deterministic model and the model including uncertainties causes a $\Delta \tau_{\mathrm{RUL}}$ RUL estimation error in the deterministic model as shown in Figure 13.

The FPGA model described in Section 3 permits considering these uncertainties by performing thousands or millions of MC simulations that accurately represent their statistics, for a more accurate RUL estimation. The results include confidence margins that are required for a reliability analysis.

## 4. Application of the Model to an Underground Cable Reliability Analysis

**Figure 14:**
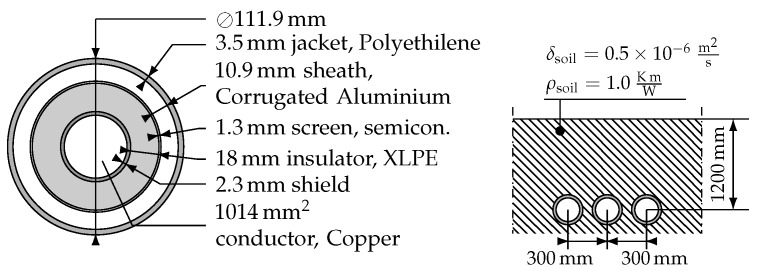
Simulated cable structure.

The architecture was tested with the cable setup shown in Figure 14, using polymer structure degradation parameters from [18], and the analysis was performed with full-year hourly data available from Red Eléctrica de España [43]. The cable is rated at 1098 A.

The cable current is measured by a hall effect sensor. The sensor is characterized by an offset calibration error $o_{\ell }$ with standard deviation $\sigma_{o}$ and a Gaussian measurement noise error η(t) with standard deviation $\sigma_{\eta }$, relative to the rated current $I_{0}$. That is, the total load estimation error $\varepsilon_{\ell }\left(t\right)$ for any sensor *ℓ* will be

(24) $$\varepsilon_{\ell }\left(t\right)=o_{\ell }+\eta \left(t\right):o_{\ell }∼N\left(0,I_{0}\sigma_{o}\right),\eta \left(t\right)∼N\left(0,I_{0}\sigma_{\eta }\right)$$

where the sensor offset calibration error $o_{\ell }$ remains constant for each sensor, while the noise error is time dependent.

**Figure 15:**
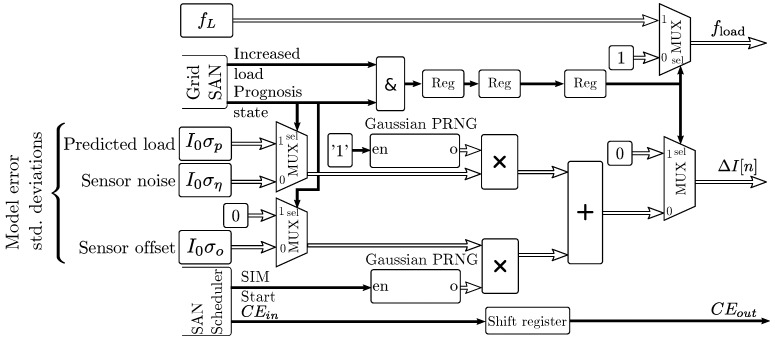
Pipelined load uncertainty model.

The sensor bias and noise models were implemented by the uncertainty generator block shown in Figure 15. The Gaussian Pseudo Random Number Generators (PRNGs) are based on a uniform PRNG and an implementation of the inverse CDF algorithm [44]. The generated numbers are multiplied by variable standard deviations defined by the simulation parameters and the current simulation state.

A prognostics prediction analysis of the cable RUL is performed at production instant $t_{0}$ = 353,000 h. The future load patterns are estimated from past statistics, and they are employed to estimate the future cable degradation paths and its RUL.

At the production instant $t_{0}$, future load patterns are preloaded into the BRAM of the current generator in Figure 12. The predicted future load estimation error $\varepsilon_{pl}\left(t\right)$ is dependent on the estimation algorithm. For this implementation, this error was modeled as $\varepsilon_{pl}\left(t\right)∼N\left(0,\sigma_{p}I_{0}\right)$. Other distributions or complex models with time-varying standard deviation can be applied depending on the grid load prognosis characteristics. As shown in Figure 15, the load uncertainty model was implemented by a multiplexer that switches between the standard deviation of the sensor noise $I_{0}\sigma_{\eta }$ and the standard deviation of the future load estimation error $I_{0}\sigma_{p}$.

**Figure 16:**
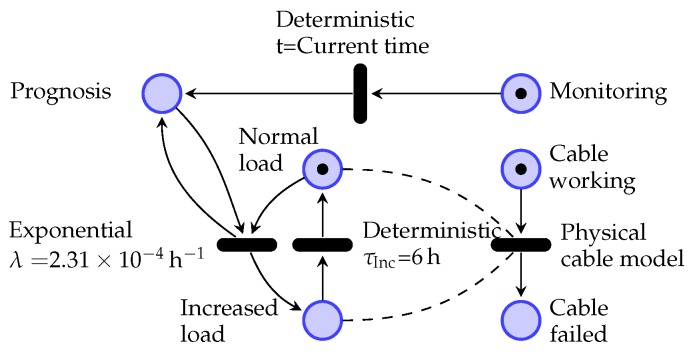
Stochastically switching grid load model implemented as use case, including the switching from monitoring to prognostics state.

Additionally, the load model for the prognosis stage includes a periodical load factor $f_{L}$. This load factor is caused by the grid switching that is simulated by the SAN simulator for the model shown in Figure 16. The random grid events were modeled as exponentially distributed events with an occurrence rate of λ=2.31e−4/h (an average period of 6 months) and a duration of $\tau_{\mathrm{Inc}}=6\;h$. The load factor increase during these intervals was modeled to be of 20% for this use case. The model in Figure 16 also includes the states for the automatic switching between diagnostics and prognosis stages.

The triggering time of activity *Physical cable model* in the SAN model is governed by the electro-thermal degradation model FPGA implementation described in Section 3. Once the model reaches the degradation limit, the activity reports the time at which the cable failed, and causes the switching to the *Cable failed* state. Notice that the behavior of the cable activity in the model is dependent on the grid state, indicated by dashed lines. These dependencies are reflected in the FPGA thermal model as connections from the load uncertainty model in Figure 15 to the SAN grid model implementation.

When the SAN model simulator reports that the *Prognosis* and *Increased load* states are marked, an increased load factor $f_{L}$ is selected by a multiplexer, which is then used as multiplier of the predicted future load values in the BRAM in Figure 12.

## 5. Results and Performance of the Architecture

**Figure 17:**
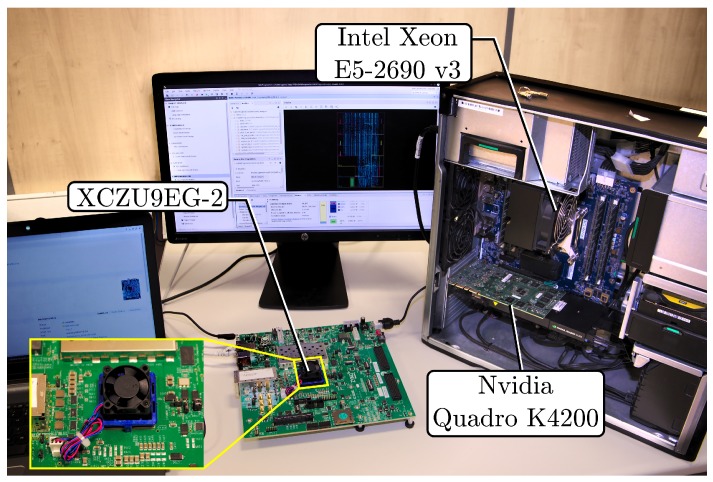
Setup used for the FPGA and software simulations.

An FPGA simulator for the SAN model in Figure 16 was connected to the described thermo-electrical degradation model. The cable parameters were imported into the FPGA design, and synthesized for a Xilinx XCZU9EG MPSoC with speed grade-2 on a Xilinx Zynq UltraScale+ MPSoC ZCU102 prototyping board at a 187MHz Programmable Logic (PL) fabric clock rate. The only purpose of the CPUs in this application is the collection of the results that are transferred by the simulator in PL to RAM by DMA. Therefore, the FPGA can be replaced by any other FPGA that meets the latency and PL resource requirements. Xilinx Vivado version 2018.1 has been employed for the synthesis, and the design was coded in VHDL 93. Figure 17 shows the setup used for both the hardware and software simulations.

**Figure 18:**
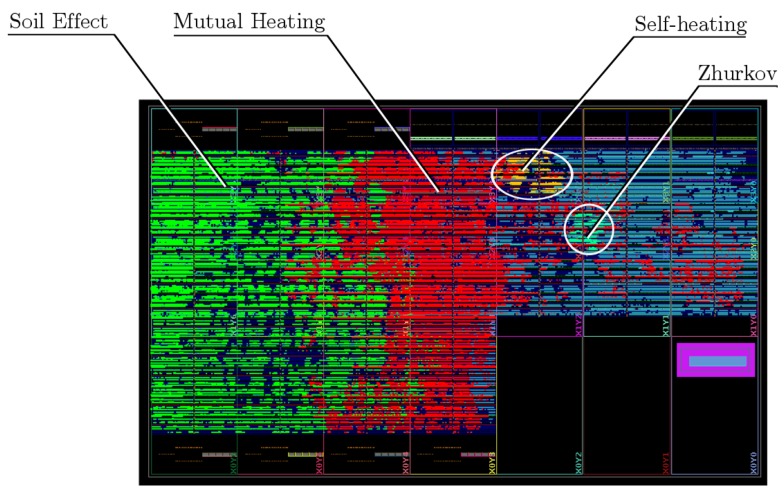
Distribution of the different model parts in the FPGA.

The FPGA design use, clock speed and synthesis timing results are displayed in Table 1, and the distribution of the separate parts in the FPGA is detailed in Figure 18. Due to the structure of the mutual and soil effect filters described in Section 3.4, most of the Configurable Logic Blocks (CLBs) of the FPGA are dedicated to the shift registers of the secondary filter stages, saving resources for the arithmetic computation. No BRAM have been used for this purpose, due to technological limitations that do not permit randomly resetting the internal register cells. In this implementation, separate filters were implemented for the soil and mutual heating processes, for debugging and testing purposes. However, both can be merged, saving large resources and reducing incremental errors.

### 5.1. Performance and Accuracy of the Architecture

**Figure 19:**
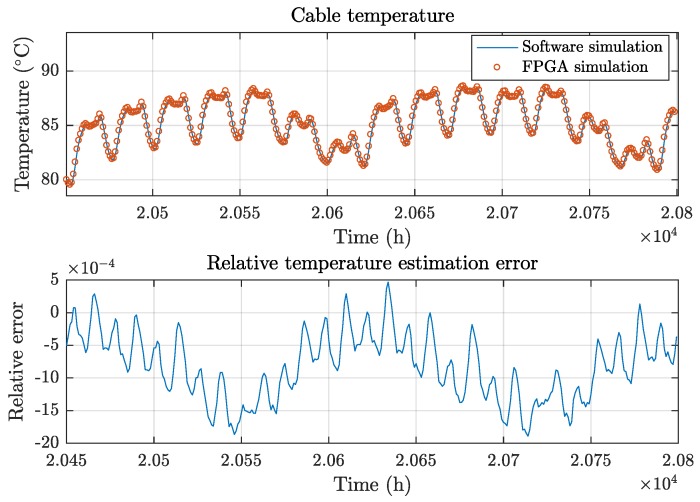
FPGA temperature estimation results compared to software simulation.

The cable was modeled to be initially as good as new with an initial degradation of γ(0)=0 and subjected to yearly load patterns obtained from [43]. The degradation level was then estimated for the beginning of the prognostics stage at $t=t_{0}$, and the simulation was run up to the cable failure time. For the validation of the architecture in software, the thermal model was adapted for an Nvidia Cuda${}^{®}$ parallel GPU computing platform and integrated within a SAN model generated within Möbius Tool [45]. Simulations were performed on a 12 core Intel${}^{®}$Xeon${}^{®}$E5-2690 v3 @ 2.60 GHz with 128 GiB RAM, and a Nvidia Quadro${}^{®}$K4200 with GPU with 1344 cores and 4 GiB memory. The resource requirements for this simulation are displayed in Table 2. Software simulations were parallelized on 12 CPU cores, and the thermal simulation was accelerated by the GPU. Each software simulation required an average of 63.45 s to perform. A single FPGA simulation took 2.29 ms to execute, performing at a rate of 435simulations/s, which is $27.7\times 10^{3}$ times faster than the equivalent computer simulation. This speed improvement permits the execution of thousands of MC simulations to analyze the impact of the uncertainties in the model. Figure 19 shows the relative error of the temperature estimations caused by the approximation described in Section 3.4 in comparison to the floating-point implementation run in software, which is also increased due to the accumulation of errors of the separate soil effect and mutual heating process filters.

The FPGA architecture permits performing hundreds of thousands or millions of MC simulations, required to represent uncertainty statistics accurately. Uncertainties include measurement errors, deviations on the expected future load or grid conditions, and model errors. Performing all the simulations in software was not feasible due to the required simulation times.

### 5.2. Diagnostics Stage

**Figure 20:**
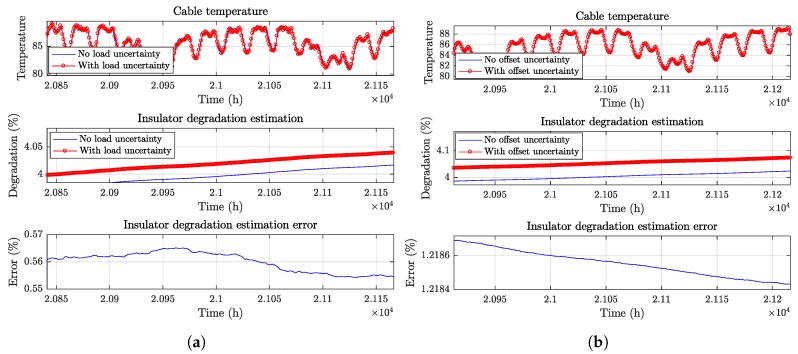
Effect on the degradation estimation by: (**a**) a 1% Gaussian standard deviation error on the load estimation; (**b**) a 0.2% load sensor bias.

Figure 20a shows the effect of stochastic load measurement errors on the estimated temperature and degradation levels, for a single simulation with $\sigma_{\eta }=1\%$ standard deviation relative to the rated current. Figure 20b shows the effect of the load sensor calibration bias, which is stochastically generated at the beginning of each simulation, with standard deviation $\sigma_{0}=0.2\%$ relative to the rated current. An offset calibration error has a greater impact on the degradation estimation, due to the error being always biased in the same direction.

**Figure 21:**
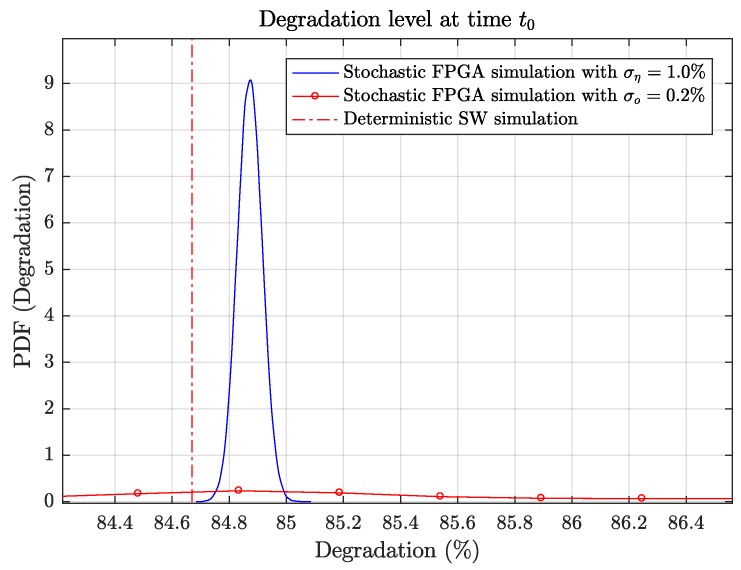
Degradation state PDF at the beginning of the prognostics stage *t* = *t*_0_.

The diagnostics stage is executed until the prognostics phase, which is performed at $t_{0}$ = 353,000 h for this use case. The software simulation estimated a degradation level of 84.67% at $t=t_{0}$. 100,000 MC simulations were performed in the FPGA, considering load sensor uncertainties. Two uncertainties were considered: (a) a sensor with a Gaussian accuracy error with $\sigma_{\eta }=1$%, and (b) sensors with an offset calibration error normally distributed with $\sigma_{o}=0.2$%. Figure 21 compares the resulting degradation probability distributions with the deterministic estimation. It can be observed that the expectation of the degradation is higher than the deterministic solution, despite the symmetric distributions of the uncertainties. This is caused by the non-linearity of the degradation function in Equation (1). It can also be noted that the impact of smaller values of $\sigma_{o}$ on the degradation is similar to the impact of larger values of $\sigma_{\eta }$. This is due to the systematic deviation caused by the offset. Additionally, the offset also increases the variance of the degradation distribution.

### 5.3. Prognosis Stage

The software simulation run without considering uncertainties predicted a cable failure at 416,388 h or a RUL of 63,388 h at the prognosis time.

When considering uncertainties after $t=t_{0}$, the future degradation of the cable was modeled to be subjected to two uncertainties: (a) errors on the predictions of the cable load $\varepsilon_{pl}\left(t\right)$; and (b) the effect of random load increases caused by unexpected grid switching.

The future load prediction error distribution depends on the prediction algorithm employed. To evaluate the sensitivity of the error, a parametric distribution was modeled $\varepsilon_{pl}\left(t\right)∼N\left(0,\sigma_{p}I_{0}\right)$.

The switching of the grid that causes random load increases is included as a SAN model shown in Figure 16 and governed by the SAN model scheduler that defines a mission time for the cable simulator, limited by the next possible state change time. During the switching periods the pipeline of the thermal model needs to be flushed empty and filled with new valid data corresponding to the new SAN model state. This process is governed by the shifted enable signals in Figure 5, Figure 8 and Figure 11a.

**Figure 22:**
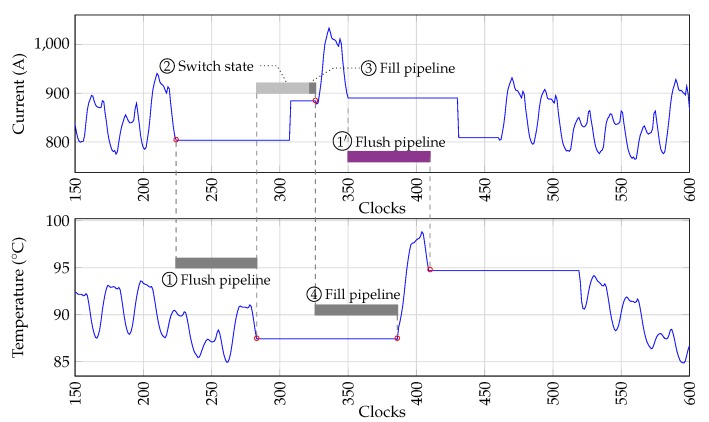
Behavior of the thermal model pipeline during a grid state change causing a load increase.

Figure 22 shows the pipeline sequence of the thermal model when the grid causes a 24 h period with an increased load factor. In this sequence the grid is initially in the *Normal load* state. Before the state change to the increased load factor, the electro-thermal model is 1 *flushed*, i.e., the pipeline continues processing until the last valid input is processed, while all inputs are flagged as invalid. At this point, the thermal model reports the current cable degradation state and the pipeline is paused until the 2 new state is defined. Notice that the cable degradation state can alter the behavior of the grid model. The pipeline is then re-enabled and continues filling the pipelines of the 3 load calculation and the 4 thermal model before the output is valid. The same process repeats at 1′ when a new state is generated, such as the return to the normal load factor state.

**Figure 23:**
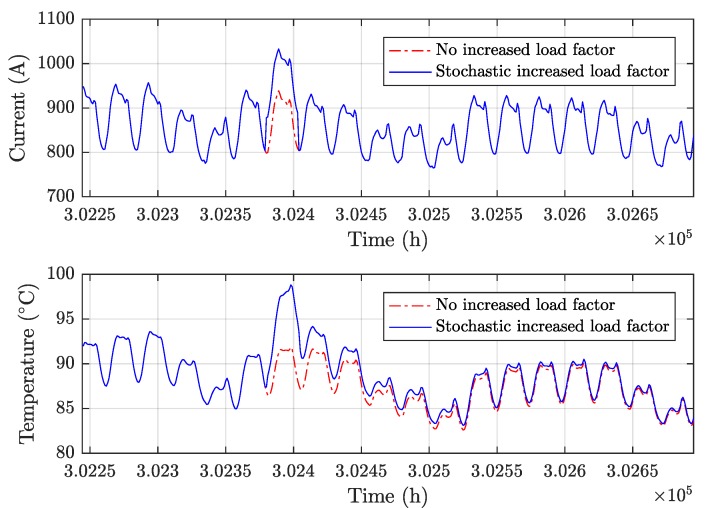
Effect of stochastic load factor increases during the prognostics stage.

Figure 23 shows the resulting impact on the cable temperature caused by the load factor increase in Figure 22.

**Figure 24:**
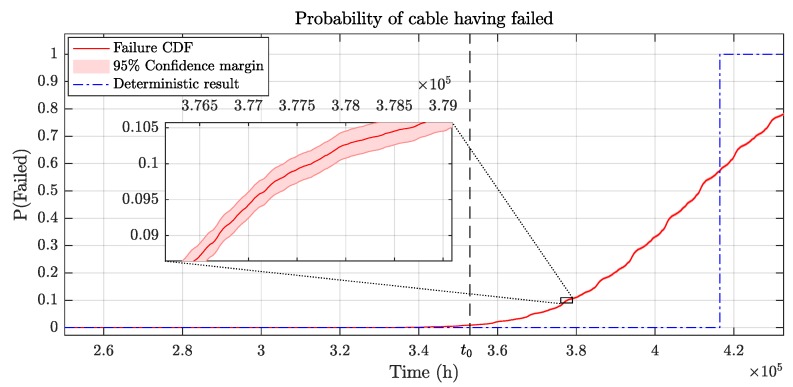
FPGA-based results considering uncertainties: $\sigma_{\eta }=1.0$%, $\sigma_{o}=0.25$%, $\sigma_{p}=5$%, λ=2.31e−4/h, and $\tau_{\mathrm{Inc}}=6h$.

Figure 24 shows a case in which a failure time prediction has been performed at time $t_{0}$ = 353,000 h. The monitoring of the cable load has been performed with a sensor with $\sigma_{\eta }=1.0\%$ and a variability in offset calibration error among sensors of $\sigma_{o}=0.25\%$. 20% load factor increases of 6h are expected every 6 months and the predicted future load uncertainty is $\sigma_{p}=5$% of the rated current. The software simulation results without uncertainties predicted a cable RUL of 63,388 h. When both diagnostics and prognosis stage uncertainties are considered with 100,000 simulations in the FPGA, the results show that there is a 10% probability that the insulator will have failed $\Delta \tau_{\mathrm{RUL}}$ = 38,748 h earlier (90% reliability time). At a 99% cable reliability requirement, the RUL of the cable is reduced by $\Delta \tau_{\mathrm{RUL}}$ = 62,556 h.

**Figure 25:**
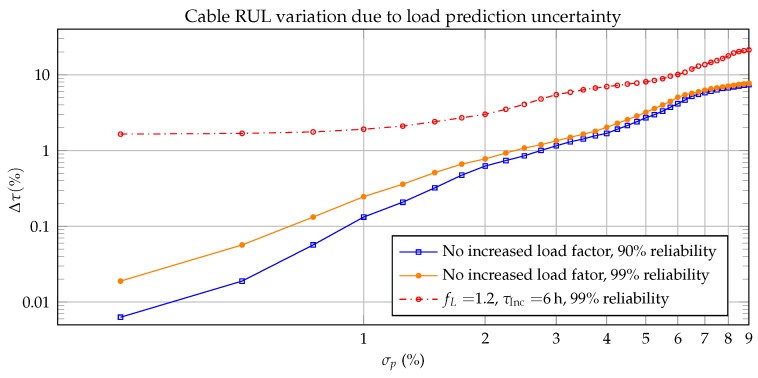
Expected RUL error due to load uncertainty during prognostics stage, relative to the predicted RUL.

To analyze the impact of load prediction uncertainties in the prognostics phase, a sensitivity analysis was performed by running the FPGA model with varying standard deviations $\sigma_{p}$ between 0% and 9%, relative to the rated current $I_{0}=1098A$. For $\sigma_{p}=2\%$, the FPGA simulation estimated a RUL of 61,896 h, 0.8% earlier than the software simulation, which can be acceptable for this application. However, when the prognostics uncertainty grows, the RUL estimation error was found to increase quasi-geometrically with the future load uncertainty, as shown in Figure 25. The results are compared to the case when stochastic grid switching with a load factor of $f_{L}=1.2\%$ is considered in the prognostics phase.

Stochastic load increases cause further degradation. The impact of the switching varies depending on the season of the year, due to the varying cable temperature, and the temperature dependent derivative in Equation (1). When the model shown in Figure 16 is considered for the load increase periods in the prognostics stage, the expected RUL drops between 1.6% and 14.7%.

## 6. Discussion

The described FPGA implementations perform in a fraction of the time required by a computer system to perform the same task and the accuracy of the thermal and degradation model implementations was found to be acceptable for its purpose. This permits the on-site evaluation of continuously evolving cable degradation including uncertainties, which was unfeasible in software, due to the long simulation times and large computer resources required.

The processing time of the implemented thermal model pipeline is deterministic for a given mission time, unless the process is interrupted by the grid SAN simulator. Therefore, the presented architecture can determine the degradation level in real time. However, it has to be noted that the simulation time can be altered by the simulation of the SAN network, due to the varying number of state transitions, and the varying number of MC simulations required for a given confidence margin of the results. Nevertheless, the simulation time can be considered negligible compared to the cable lifetime, for practical purposes.

For this implementation, a Zhurkov degradation model has been adopted but a similar approach can be followed for an Arrhenius model if required [18].

During the prognosis stage the load uncertainty error was modeled as a Gaussian process. This error depends on the load forecasting algorithm employed and the probability distribution could be time-varying for long-term predictions. However, adapting the uncertainty generator to other load forecasting algorithms should be straightforward as far as a probability distribution function of the prediction algorithm can be obtained.

The objective of the article was to demonstrate the use of a hardware-based thermal model for the accelerated estimation of the cable reliability. In the designed use case, load measurement, grid switching, and future load uncertainties have been considered. However, different time-varying and uncertain factors can be included in the uncertainty generator, including varying degradation model parameters, apart from the uncertainties modeled in this use case. To adapt the architecture for different grids it is possible to include other factors such as the yearly thermal behavior, humidity of the soil and variation of its resistivity can greatly affect the thermal behavior and the degradation of the cable [46]. Uneven terrains can also be considered by simulating the multiple sections separately.

## 7. Conclusions

The article proposes a new hardware architecture that permits performing an on-site reliability analysis and prognosis of underground cables based on past load measurements and future load predictions.

New pipelined architectures have been proposed for the simulation of the non-linear functions. The architecture permits considering uncertainties and results are obtained within a fraction of the time required by software simulations. The effects of load uncertainties and grid switching events have been analyzed experimentally by the simulation of the effects on a specific underground cable system. Therefore, the architecture is deemed suitable for use in live monitoring applications.

## Figures and Tables

**Table 1:** FPGA resource use, timing, and thermal characteristics of the implementation.

**FPGA Device**			**(XCZU9EG-2)**			
**Time and Thermal Values**			**Value**			
PL Clock			187MHz			
Worst Negative Slack (WNS)			0.090 ns			
Worst Hold Slack (WHS)			0.010 ns			
Worst PulseWidth Slack (WPWS)			3.750 ns			
Thermal Margin@25.0 °C			68.4 °C			
**Resource**	**Mutual Heating**	**Soil Effect**	**Self-Heating**	**Zhurkov**	**Total**	**% of Total**
LUT	47,766	72,359	1833	1296	152,830	55.76%
Registers	48,451	37,303	911	266	114,947	20.97%
BRAM	0	0	1	1	35	3.84%
DSP	1374	273	0	0	1663	65.99%

**Table 2:** Simulation timing and resource use.

	Software Simulation	FPGA Simulation
Number of Simulations	12	100,000
Total Simulation Time	761.475 s	229.71 s
Single simulation Time	63.456 s	2.297 ms
RAM Usage	9.17 GiB	1.53 MiB
GPU Memory Usage	1.35 GiB	0
No. CPU Cores	12	0
No. GPU Cores	1344	0

## Nomenclature

The following symbols are used in this manuscript:

ϑ	Absolute temperature (K)
$\tau_{d}$	Latency of a block (clock)
θ	Temperature (${}^{\circ }C$)
$\tau_{Mp}$	Maximum block latency of a pipeline (clock)
*E*	Electric field (V $m^{-1}$)
$\tau_{dp}$	Latency of the pipeline (clock)
$P_{D}$	Design failure probability of the insulator
μ	Marking of the SAN model
*D*	Enlargement factor
$pl_{i}$	ith place in a SAN model
$\alpha_{\tau ,0}$	Scale parameter
$ac_{i}$	ith activity in a SAN model
$\beta_{t}$	Shape parameter of the Weibull probability distribution of the time to failure of the cable
$\mu_{i}$	Marking of the ith place in a SAN model
*w*	Activation energy of the insulator material (J ${\mathrm{mol}}^{-1}$)
$t_{0}$	Production instant (s)
χ	Structural parameter of the insulator material (J m ${\mathrm{mol}}^{-1}$ $V^{-1}$)
n	Discrete-time index
*R*	Universal gas constant ($J\;{\mathrm{mol}}^{-1}$ $K^{-1}$)
ΔW	Dissipated power increment (W $m^{-1}$)
τ(θ,E)	Lifetime of insulator under temperature θ and electric field *E* (s)
$g_{pk}$	Unit step response of the mutual heating process (${}^{\circ }C$ m $W^{-1}$)
$\theta_{i}$	Temperature at time interval *i* (${}^{\circ }C$)
$g_{soil}$	Unit step response of the soil effect (${}^{\circ }C$ m $W^{-1}$)
$E_{i}$	Electric field at time interval *i* (V $m^{-1}$)
${\hat{g}}_{pk}$	Approximate step response of the mutual heating process (${}^{\circ }C$ m $W^{-1}$)
$\Delta \gamma_{i}$	Degradation increment at time interval *i* (p.u.)
*L*	Time index at which the step response is segmented for its approximation
$\theta_{s}$	Temperature rise due to the self-heating (${}^{\circ }C$)
*M*	Time index at which the step response is segmented for its approximation
$\theta_{e}$	Temperature rise due to the soil effect (${}^{\circ }C$)
*q*	Number of sub-filter stages in the second segment of the approximate thermal process
$\theta_{pk}$	Temperature rise due to the mutual heating (${}^{\circ }C$)
$s_{i}$	Output of the ith stage in the second segment of the approximate thermal process
α(t)	Attainment factor
CE	Clock enable signal
$T_{x}$	Thermal resistance of layer *x* in an equivalent thermal circuit (K m $W^{-1}$)
$\tau_{s}$	Sampling interval (s)
$Q_{x}$	Thermal capacitance of layer *x* in an equivalent thermal circuit (J $m^{-1}$ $K^{-1}$)
$\lambda_{1,m}$	Sheath loss factor for the middle cable
H(s)	Transfer function for the self-heating process
$\lambda_{2,m}$	Armor loss factor for the middle cable
Ei	Exponential integral
$\lambda_{1,si}$	Sheath loss factor for the ith adjacent cable
$D_{e}$	External cable diameter (m)
$\lambda_{2,si}$	Armor loss factor for the ith adjacent cable
$\delta_{s}$	Thermal diffusivity of the soil ($m^{2}$ $s^{-1}$)
$f_{L}$	Load factor
$\rho_{s}$	Thermal resistivity of the soil (K m $W^{-1}$)
$\rho_{c}$	Electrical resistivity of the conductor (Ωm)
$L_{d}$	Depth of the cable in the soil (m)
$\alpha_{c}$	Temperature coefficient of the resistivity of the conductor ($K^{-1}$)
$W_{t}$	Total power dissipated within the cable (W $m^{-1}$)
$\tau_{RUL}$	Predicted remaining useful life (s)
$d_{pk}$	Distance to the adjacent cable causing the mutual heating (m)
$\Delta \tau_{RUL}$	Difference in the predicted remaining useful life (s)
$d_{pk}^{\prime }$	Distance to the mirror of the adjacent cable causing the mutual heating (m)
$\varepsilon_{\ell }$	Load estimation error (A)
$o_{\ell }$	Calibration offset error (A)
η	Load measurement noise error (A)
$I_{0}$	Rated current (A)
$\sigma_{o}$	Relative standard deviation of the calibration offset
γ	Degradation of the insulator (p.u.)
$\sigma_{\eta }$	Relative standard deviation of the noise
$\varepsilon_{p\ell }$	Predicted future load estimation error (A)
*t*	Time (s)
